# Causal links between serum micronutrients and epilepsy: a Mendelian randomization analysis

**DOI:** 10.3389/fneur.2024.1419289

**Published:** 2024-07-15

**Authors:** Haohao Chen, Zequn Zheng, Xiaorui Cai, Fenfei Gao

**Affiliations:** ^1^Department of Pharmacy, Shantou University Medical College, Shantou, Guangdong Province, China; ^2^Department of Pharmacy, The First Affiliated Hospital of Shantou University Medical College, Shantou, Guangdong Province, China; ^3^Department of Cardiology, The First Affiliated Hospital of Shantou University Medical College, Shantou, Guangdong, China; ^4^Clinical Research Center, The First Affiliated Hospital of Shantou University Medical College, Shantou, Guangdong, China; ^5^Ningbo Institute of Innovation for Combined Medicine and Engineering, Ningbo Medical Center Lihuili Hospital, Ningbo University, Ningbo, Zhejiang, China; ^6^Department of Pharmacy, The Affiliated Cancer Hospital of Shantou University Medical College, Shantou, Guangdong, China

**Keywords:** epilepsy, micronutrients, Mendelian randomization, causal association, genome-wide association studies

## Abstract

**Background:**

Micronutrient levels play a critical role in epilepsy. This study investigates the impact of micronutrient levels on epilepsy via Mendelian randomization (MR).

**Methods:**

A two-sample MR framework evaluated the genetic association between 15 serum micronutrients and epilepsy phenotypes. The analysis included calcium, iron, zinc, selenium, copper, magnesium, potassium, folate, vitamins B6, B12, C, D, E, retinol, and carotene against all epilepsy, generalized epilepsy, childhood absence epilepsy (CAE), juvenile absence epilepsy (JAE), juvenile myoclonic epilepsy (JME), generalized tonic–clonic seizures alone and with spike–wave electroencephalography (GTCS), and various focal epilepsy phenotypes [with hippocampal sclerosis (HS), lesions other than HS, lesion-negative]. The random-effects inverse-variance weighted (IVW) model was the primary method used, supported by heterogeneity and pleiotropy assessments. Multivariable Mendelian randomization analyses (MVMR) were used to identify micronutrients that are significantly causally associated with different epilepsy subtypes and to confirm the most potential causal risk factors for these subtypes.

**Results:**

Zinc conferred an increased risk of focal epilepsy with HS (OR = 1.01; *p* = 0.045). Carotene was similarly linked to higher risks of lesion-negative cases (OR = 1.129; *p* = 0.037). Conversely, vitamin B6 was associated with reduced risks of focal epilepsy with HS (OR = 0.949; *p* = 0.020), and vitamin D was linked to decreased risks of both CAE (OR = 0.976, 95% CI: 0.959–0.993, *p* = 0.006) and JAE (OR = 0.986, 95% CI: 0.973–0.999, *p* = 0.032). These associations were robust, showing minimal heterogeneity and no evidence of pleiotropy across various sensitivity analyses. After adjustment using MVMR, significant causal relationships between vitamin D and both CAE and JAE remained. Furthermore, the causal relationship between zinc and vitamin B6 on focal epilepsy with HS became non-significant, while carotene shifted from a risk factor to a protective factor for focal epilepsy lesion-negative after adjusting for vitamin D.

**Conclusion:**

MR estimates provide robust evidence for the causal effects of vitamin D on reducing the risk of CAE, and JAE, which might provide alternative treatment strategies.

## 1 Introduction

Epilepsy stands as a predominant neurological disorder worldwide, affecting individuals across all age groups. With a point prevalence of 6.4 per 1,000 individuals and an incidence rate of 61.4 per 100,000 person-years, it impacts more than 70 million people globally, making it a significant public health concern (1, 2). Despite pharmacotherapy being the cornerstone of epilepsy management, the phenomenon of pharmacoresistance is evident in about one-third of the patient population, necessitating the exploration of alternative therapeutic avenues and the identification of modifiable risk factors for effective prevention and treatment (3, 4).

Recent investigations have shed light on the intricate link between epilepsy and the dysregulation of micronutrients, with elements such as copper, iron, manganese, zinc, selenium, phosphorus, magnesium, and vitamins D, vitamin B6, and folate being implicated in the modulation of the disease’s progression (5–8). However, the evidence has predominantly derived from animal studies, remains preliminary, and is often marred by the inherent limitations of observational research, such as residual confounding and reverse causality, thus calling for a more rigorous methodological approach to ascertain these relationships.

MR, leveraging genetic variants such as SNPs as instrumental variables (IVs), offers a robust method to ascertain causality between exposures and outcomes with reduced biases, including reverse causation and confounding common in observational studies (9, 10). This study examines the effect of micronutrients (e.g., essential minerals, vitamins B6, B12, C, D, E, retinol, and carotene) on various epilepsy outcomes, ranging from general to specific types such as CAE, JAE, JME, GTCS, and both generalized and focal epilepsies, with a focus on differentiating those with and without specific brain lesions.

By leveraging the MR framework, our study embarks on elucidating the potential causal links between various micronutrients and epilepsy, aiming to contribute valuable insights into the pathophysiological underpinnings and offering new perspectives on the management and prevention of this complex neurological disorder (11–13).

## 2 Materials and methods

### 2.1 Study overview

**Figure 1:**
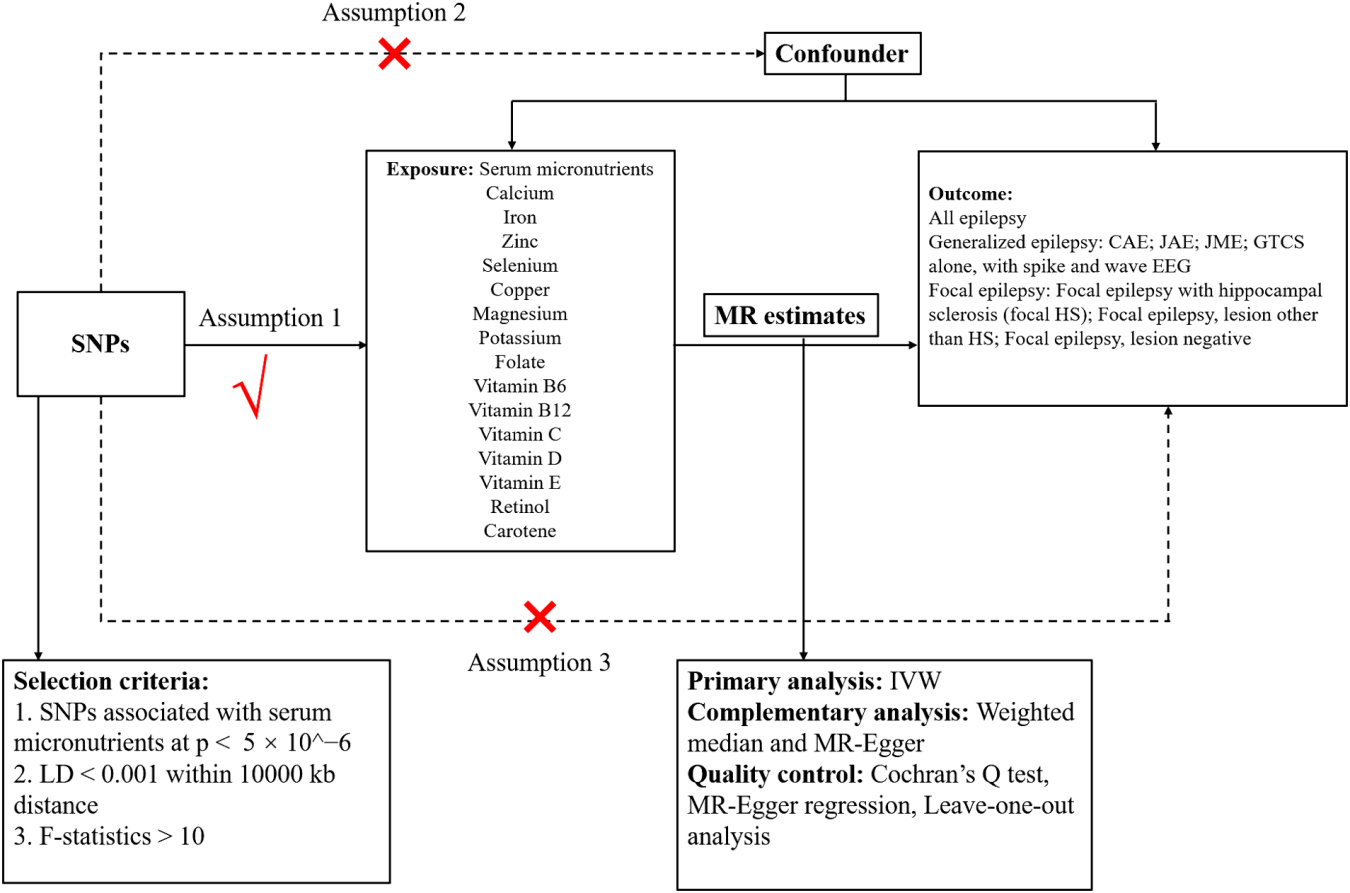
Schematic diagram showing the assumptions of Mendelian randomization analysis of serum micronutrients (calcium, iron, zinc, selenium, copper, magnesium, potassium, folate, and vitamin B6, vitamin B12, vitamin C, vitamin D, vitamin E, retinol, and carotene) on epilepsy [all epilepsy, generalized epilepsy, CAE, JAE, JME, GTCS alone and with spike and wave electroencephalography, focal epilepsy, focal epilepsy with hippocampal sclerosis (focal epilepsy with HS); focal epilepsy lesions other than HS; focal epilepsy lesion-negative]. SNPs, single-nucleotide polymorphisms; CAE, childhood absence epilepsy; JAE, juvenile absence epilepsy; JME, juvenile myoclonic epilepsy; GTCS, generalized epilepsy with tonic–clonic seizures; LD, linkage disequilibrium; IVW, inverse-variance weighted.

We performed a two-sample MR analysis to explore the causal links between 15 micronutrients and various epilepsy phenotypes. The selection of these micronutrients was informed by their potential biological relevance to epilepsy pathogenesis. We aimed to determine the causal effects of specific exposures on outcomes by utilizing summary statistics from GWAS derived from two independent datasets through two-sample MR analysis. The validity of this MR approach was contingent upon three core assumptions: (1) instrument strength: the genetic variants selected as instruments must have a robust association with the exposure of interest. This ensures that the genetic instruments are valid proxies for the exposure. (2) Exclusivity of pathway: the genetic instruments should affect the outcome exclusively through their relationship with the exposure, thereby establishing a direct causal link between the exposure and the outcome. (3) Instrument Independence: The genetic instruments must be free from confounding variables that could influence both the exposure and the outcome, thereby precluding alternative causal pathways. Figure 1 illustrates the study design, encapsulating the analytical process and the systematic approach employed to ascertain the causal relationships.

### 2.2 GWAS summary statistics

GWAS data on micronutrients was sourced from the IEU Open GWAS database^1^ and is summarized in Table 1, with comprehensive details provided in Supplementary Tables S1–S15. GWAS summary statistics on epilepsy were acquired from the 2018 dataset of the ILAE Consortium (14). The dataset was categorized by epilepsy type and meticulously assessed following ILAE guidelines, incorporating electroencephalography, imaging, and clinical history data. Analyzed epilepsy categories encompassed all epilepsy cases, generalized epilepsy, and focal epilepsy, with specific subcategories such as CAE and JAE (Table 2). Fixed-effect transethnic meta-analyses were conducted to calculate *Z* scores, which were then transformed into *β* coefficients and standard errors (SE) (15).

**Table 1:** Detailed characteristics of GWAS data sets employed as instrumental variables in Mendelian randomization analysis.

ID	Trait	First author	Consortium	Sex	PMID	Population	Sample size	Year	SNPs
ebi-a-GCST90025990	Calcium	Cantin Baron	NA	NA	34,226,706	European	400,792	2021	311
ukb-b-20447	Iron	Ben Elsworth	MRC-IEU	Males and Females	NA	European	64,979	2018	14
ieu-a-1079	Zinc	David M. Evans	NA	Males and Females	23,720,494	European	2,603	2013	8
ieu-a-1077	Selenium	David M. Evans	NA	Males and Females	23,720,494	European	2,603	2013	6
ieu-a-1073	Copper	David M. Evans	NA	Males and Females	23,720,494	European	2,603	2013	6
ukb-b-7372	Magnesium	Ben Elsworth	MRC-IEU	Males and Females	NA	European	64,979	2018	19
ukb-b-17881	Potassium	Ben Elsworth	MRC-IEU	Males and Females	NA	European	64,979	2018	16
ukb-b-11349	Folate	Ben Elsworth	MRC-IEU	Males and Females	NA	European	64,979	2018	15
ukb-b-7864	Vitamin B6	Ben Elsworth	MRC-IEU	Males and Females	NA	European	64,979	2018	18
ukb-b-19524	Vitamin B12	Ben Elsworth	MRC-IEU	Males and Females	NA	European	64,979	2018	10
ukb-b-19390	Vitamin C	Ben Elsworth	MRC-IEU	Males and Females	NA	European	64,979	2018	11
ebi-a-GCST90025967	Vitamin D	Cantin Baron	NA	NA	34,226,706	European	418,691	2021	152
ukb-b-6888	Vitamin E	Ben Elsworth	MRC-IEU	Males and Females	NA	European	64,979	2018	12
ukb-b-17406	Retinol	Ben Elsworth	MRC-IEU	Males and Females	NA	European	62,991	2018	9
ukb-b-16202	Carotene	Ben Elsworth	MRC-IEU	Males and Females	NA	European	64,979	2018	16

**Table 2:** Data sources of epilepsy.

Phenotype	Sample size		Population (%European)
	Cases, *n*	Controls, *n*	
All epilepsy	15,212	29,677	95.5
Generalized epilepsy	3,769	29,677	98.3
Focal epilepsy	9,671	29,677	94
Childhood absence epilepsy	778	24,218	100^a^
Juvenile absence epilepsy	415	24,218	100^a^
Juvenile myoclonic epilepsy	1,177	24,218	100^a^
GTCS alone, with spike and wave EEG	225	24,218	100^a^
Focal epilepsy with HS	709	24,218	100^a^
Focal epilepsy lesions other than HS	2,751	24,218	100^a^
Focal epilepsy lesion-negative	2,660	24,218	100^a^

SNP, Single-nucleotide polymorphism.

a GWAS of specific syndromes of genetic generalized epilepsy and phenotypes of focal epilepsy were limited to European ancestry.

### 2.3 Selection of instrumental variables

For the IVs in this MR study, we utilized summary GWAS data on serum micronutrients from a recent publication (16), with updates on calcium and vitamin D from the latest findings (17). Our selection process for instrumental SNPs associated with micronutrients involved rigorous quality control steps. Initially, we identified SNPs strongly linked to micronutrients (*p* < 5 × 10^−6^) using clumping criteria of linkage disequilibrium (LD) *r*^2^ > 0.001 within a 10,000-kilobase window, referencing the 1,000 Genomes European panel. SNPs not found in the epilepsy outcome dataset were substituted with proxy SNPs from SNiPA at the website https://snipa.org/snipa3/?task=proxy_search, adhering to an LD *r*^2^ > 0.8 threshold. SNPs lacking appropriate proxies were discarded. We computed the *F* statistic for each SNP to assess instrument strength, aiming for an *F* statistic above 10 to avoid weak instrument bias, following the method outlined by Pierce (18).

### 2.4 MR estimates

Our primary MR analysis utilized the random-effects IVW method to amalgamate individual SNP-based causal estimates into an overall exposure-outcome effect. This method was chosen for its conservative estimation approach, particularly in accounting for pleiotropy (19). The analysis adhered to a *p*-value threshold of <0.05 to determine statistical significance. To verify the IVW results, we compared them with other MR methods, such as the weighted median and MR-Egger, provided a sufficient number of SNPs were available for analysis (20). Multivariable Mendelian randomization (MVMR) is an extension of univariable MR that accounts for pleiotropy among multiple traits (21). Adjustment for micronutrients was performed using multivariable Mendelian randomization (MVMR) to determine if there was a synergistic effect among the micronutrients that were significantly causal for epilepsy subtypes in the two-sample Mendelian randomization analysis (22).

### 2.5 Quality control of MR estimation

Heterogeneity in the IVW model was assessed using the Cochran *Q* test and *I*^2^ statistics, while the Rucker *Q*^0^ test was used to evaluate heterogeneity in the MR-Egger model. The difference between *Q* and *Q*^0^ was calculated to ascertain the more suitable model, with a negligible *Q*–*Q*^0^ difference (*p* > 0.05) favoring the IVW model for better fit. Directional pleiotropy was examined using the MR-Egger intercept (*p* > 0.05). To ensure the robustness of our findings, a leave-one-out sensitivity analysis was conducted to identify any SNP excessively influencing the causal estimate (23–25). To affirm our causal conclusions, we ensured (a) consistent results across MR methods, (b) non-significant directional pleiotropy in MR-Egger intercepts, (c) congruence in heterogeneity assessments between Cochran’s *Q* test and Rucker’s *Q*^0^, and (d) no undue global influence from any single SNP in the leave-one-out analysis.

MR analyses were conducted in R (version 4.3.3; R Foundation for Statistical Computing, Vienna, Austria) with the “TwoSampleMR” packages.

## 3 Results

### 3.1 Overview of study results

Our investigation into the causal connections between 15 serum micronutrients and epilepsy utilized a stringent SNP selection process, resulting in 6–311 SNPs per micronutrient phenotype, with *F* statistics ranging from 18.08 to 3545.25, as detailed in Supplementary Tables S1–S15. Our primary analysis unveiled six statistically significant causal associations (*p* < 0.05), including zinc on focal epilepsy with HS, carotene on focal epilepsy lesions other than HS, carotene on focal epilepsy lesion-negative, vitamin B6 on focal epilepsy with HS, vitamin D on CAE, and vitamin D on JAE.

### 3.2 Causal effects of micronutrients on epilepsy

**Figure 2:**
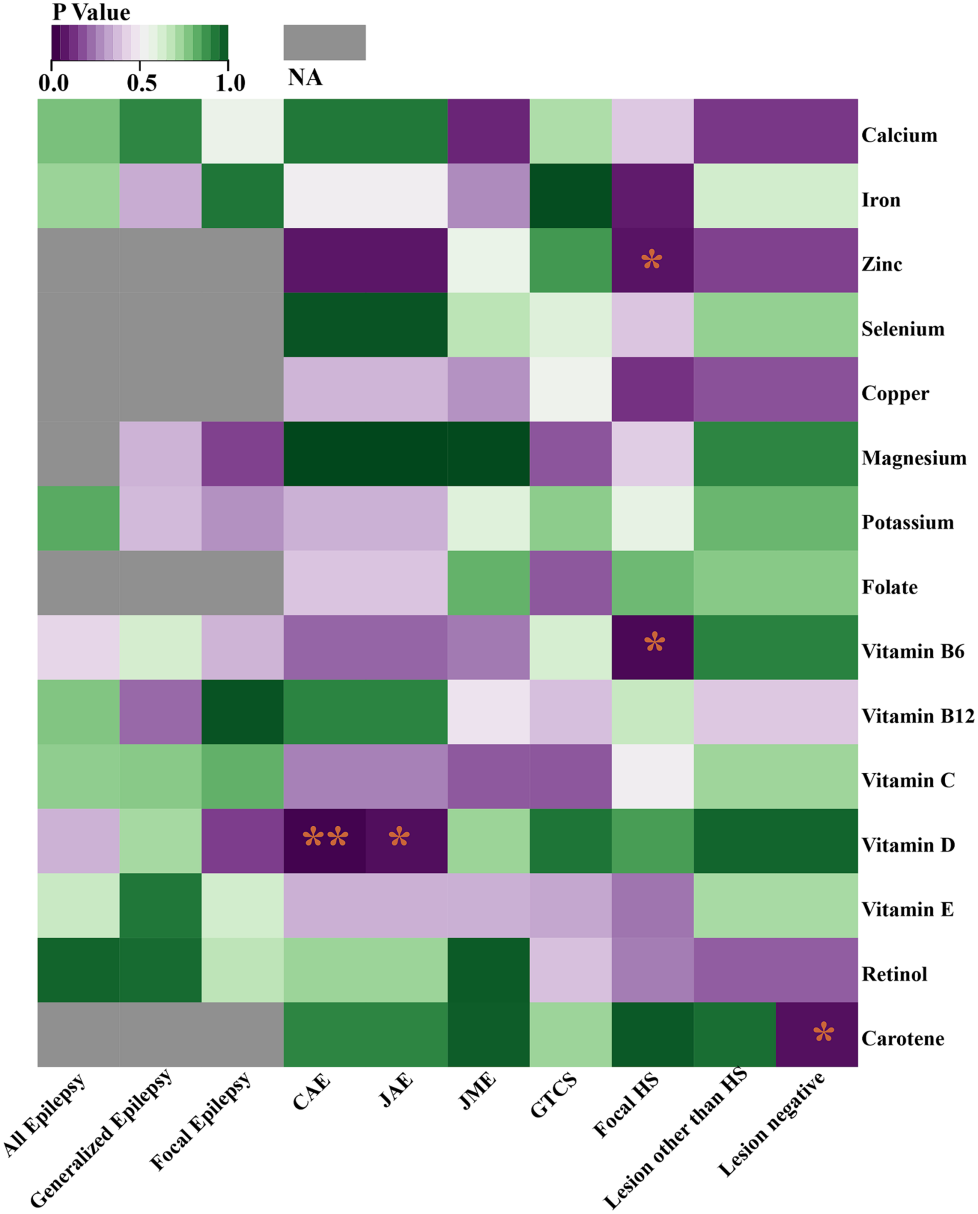
Heat map represents the causal associations between 15 serum micronutrients and various forms of epilepsy as identified through the IVW method. *p < 0.05; **p < 0.01; NA, Not available.

**Figure 3:**
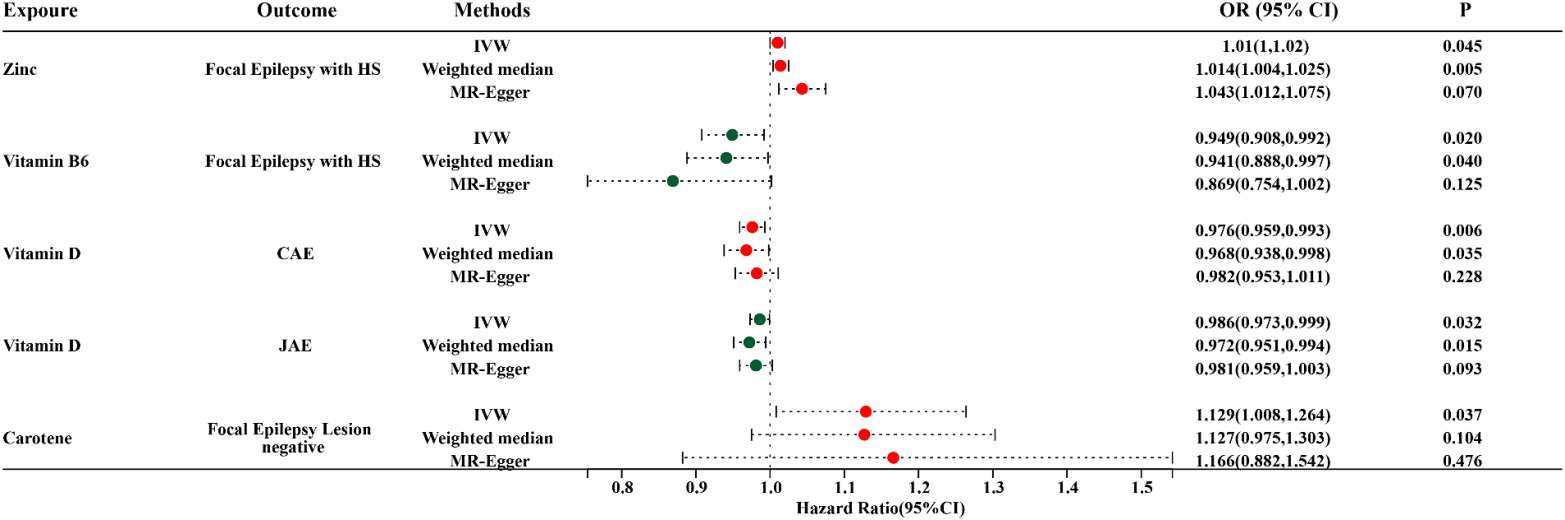
Forest plots for IVW, Weighted median, and MR-Egger MR methods account for the association between specific micronutrients (zinc, vitamin B6, vitamin D, and carotene) and different epilepsy phenotypes (focal epilepsy with HS, CAE, JAE, focal epilepsy lesion-negative). HS, hippocampal sclerosis; CAE, childhood absence epilepsy; JAE, juvenile absence epilepsy; OR, odds ratio; 95% CI, 95% confidence interval.

Through the IVW model analysis, we identified that zinc, carotene, vitamin B6, and vitamin D significantly influenced epilepsy outcomes, including focal epilepsy with HS, focal epilepsy lesions other than HS, focal epilepsy lesion-negative, CAE, and JAE. Notably, zinc was associated with an increased risk of focal epilepsy with HS (OR = 1.010, 95% CI: 1.000–1.020, *p* = 0.045). Carotene showed a connection to a higher risk of focal epilepsy in both lesions other than HS (OR = 1.129, 95% CI: 1.008–1.264, *p* = 0.037) and lesion-negative cases (OR = 1.129, 95% CI: 1.008–1.264, *p* = 0.037). In contrast, vitamin B6 was linked to reduced risk of focal epilepsy with HS (OR = 0.949, 95% CI: 0.908–0.992, *p* = 0.020), and vitamin D was linked to decreased risks of both CAE (OR = 0.976, 95% CI: 0.959–0.993, *p* = 0.006) and JAE (OR = 0.986, 95% CI: 0.973–0.999, *p* = 0.032). Other micronutrients, including calcium, iron, selenium, copper, magnesium, potassium, folate, vitamins B12, C, E, and retinol, did not exhibit significant causal relationships in our MR analysis for epilepsy phenotypes. The causal associations between 15 serum micronutrients and various forms of epilepsy, as identified through the IVW method, are shown in Figure 2, Forest plots for IVW, weighted median, and MR-Egger methods account for the association between specific micronutrients (zinc, vitamin B6, vitamin D, carotene) and different epilepsy phenotypes (focal epilepsy with HS, CAE, JAE, focal epilepsy lesions other than HS, focal epilepsy lesion-negative), and are showed in Figure 3 and Table 3, with comprehensive details provided in Supplementary Table S16.

**Table 3:** Mendelian randomization estimates of the causal relationships between micronutrients and epilepsy derived from various models.

Exposures	Outcomes	SNPs (*n*)	Inverse variance weighted		Weighted median		MR-Egger	
			OR (95% CI)	*p*	OR (95% CI)	*p*	OR (95% CI)	*p*
Zinc	Focal epilepsy with HS	5	1.01 (1, 1.02)	0.045	1.014 (1.004, 1.025)	0.005	1.043 (1.012, 1.075)	0.070
Vitamin B6	Focal epilepsy with HS	6	0.949 (0.908, 0.992)	0.020	0.941 (0.888, 0.997)	0.040	0.869 (0.754, 1.002)	0.125
Vitamin D	CAE	101	0.976 (0.959, 0.993)	0.006	0.968 (0.938, 0.998)	0.035	0.982 (0.953, 1.011)	0.228
Vitamin D	JAE	101	0.986 (0.973, 0.999)	0.032	0.972 (0.951, 0.994)	0.015	0.981 (0.959, 1.003)	0.093
Carotene	Focal epilepsy lesion-negative	3	1.129 (1.008, 1.264)	0.037	1.127 (0.975, 1.303)	0.104	1.166 (0.882, 1.542)	0.476

We used MVMR to analyze the causal relationships between zinc, vitamin B6, carotene, and vitamin D with epilepsy subtypes: focal epilepsy with HS, focal epilepsy lesion-negative, CAE, and JAE. This analysis aimed to determine if there were mutual influences among these micronutrients. After adjustment, the significant causal relationships between vitamin D on both CAE and JAE remained unchanged. For CAE, the adjusted *p*-values and *β*-values were: zinc (*p* = 0.004, *β* = 0.974), carotene (*p* = 0.004, *β* = 0.975), and vitamin B6 (*p* = 0.004, *β* = 0.975). For JAE, the adjusted *p*-values and *β*-values were: zinc (*p* = 0.029, *β* = 0.985), carotene (*p* = 0.021, *β* = 0.985), and vitamin B6 (*p* = 0.016, *β* = 0.985). After adjusting for carotene, vitamin B6, and vitamin D, the causal relationship between zinc and focal epilepsy with HS was no longer significant, with *p*-values of 0.057, 0.114, and 0.837, respectively. The relationship between vitamin B6 and focal epilepsy with HS was also not significant after adjustment. For carotene and focal epilepsy lesion-negative, the adjusted *p*-values and *β*-values were: zinc (*p* = 0.255), vitamin B6 (*p* = 0.015, *β* = 1.190), and vitamin D (*p* = 0.044, *β* = 0.992). Significance and directionality were only maintained for vitamin B6. After adjusting for vitamin D, carotene changed from being a risk factor to a protective factor. More detailed information is provided in Table 4.

**Table 4:** Multivariable Mendelian randomization results of specific micronutrients and epilepsy subtypes.

Exposure	Potential confounding factors	Outcome	OR (95% CI)	*p*
Zinc	None	Focal epilepsy with HS	1.010 (1.000, 1.020)	0.045
	Carotene		1.010 (1.000, 1.020)	0.057
	Vitamin B6		1.008 (0.998, 1.019)	0.114
	Vitamin D		0.998 (0.976, 1.020)	0.837
Carotene	None	Focal epilepsy lesion-negative	1.129 (1.008, 1.264)	0.037
	Zinc		1.089 (0.940, 1.262)	0.255
	Vitamin B6		1.190 (1.034, 1.368)	0.015
	Vitamin D		0.922 (0.852, 0.998)	0.044
Vitamin B6	None	Focal epilepsy with HS	0.949 (0.908, 0.992)	0.020
	Zinc		0.943 (0.879, 1.011)	0.100
	Carotene		0.944 (0.886, 1.005)	0.072
	Vitamin D		0.987 (0.942, 1.034)	0.593
Vitamin D	None	Childhood absence epilepsy	0.976 (0.959, 0.993)	0.006
	Zinc		0.974 (0.957, 0.992)	0.004
	Carotene		0.975 (0.959, 0.992)	0.004
	Vitamin B6		0.975 (0.959, 0.992)	0.004
Vitamin D	None	Juvenile absence epilepsy	0.986 (0.973, 0.999)	0.032
	Zinc		0.985 (0.972, 0.998)	0.029
	Carotene		0.985 (0.973, 0.998)	0.021
	Vitamin B6		0.985 (0.972, 0.997)	0.016

### 3.3 Sensitive analyses

**Figure 4:**
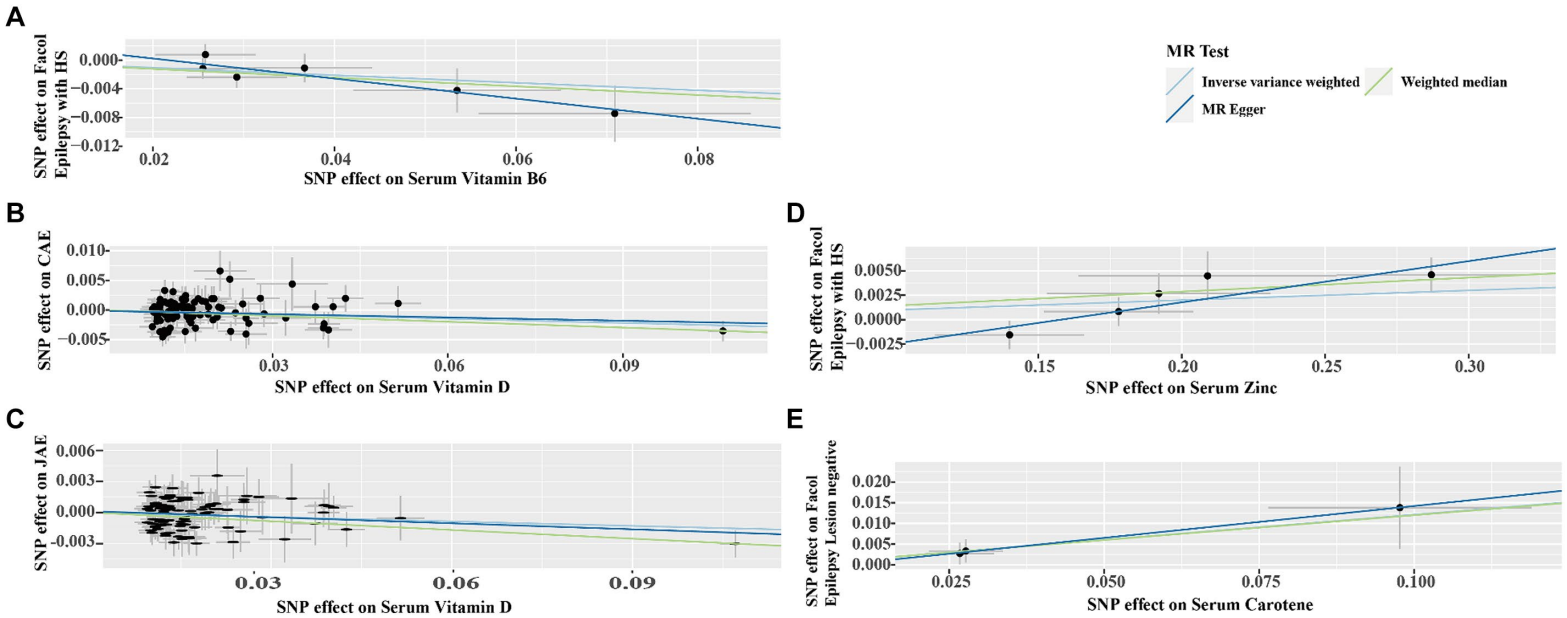
Scatter plots demonstrating the strong genetic correlations between serum micronutrients (zinc, carotene, vitamin B6, and vitamin D) and different epilepsy forms (focal epilepsy with HS, CAE, JAE, focal epilepsy lesion-negative). Each plot features single nucleotide polymorphisms (SNPs) linked to the micronutrient levels, depicted as black dots. The error bars represent the standard error associated with each SNP’s effect on the micronutrient (horizontal axis) and the specific epilepsy type (vertical axis). The line slope in each plot indicates the magnitude and direction of the causal relationship, as established by IVW, Weighted Median, and MR-Egger methods. **(A)** Serum vitamin B6 on focal epilepsy with HS; **(B)** Serum vitamin D on CAE; **(C)** Serum vitamin D on JAE; **(D)** Serum Zinc on Focal Epilepsy with HS; **(E)** Serum carotene on focal epilepsy lesion-negative. CAE, childhood absence epilepsy; JAE, juvenile absence epilepsy; HS, hippocampal sclerosis.

Cochran’s *Q* test detected no heterogeneity in the IVW model for the specific exposure and outcomes described in Section 3.2, with *p-*values ranging from 0.163 to 0.963 (Table 5). The Rucker framework highlighted no heterogeneity, indicating the IVW model is suitable for analyzing the causal relationship. The MR-Egger intercept indicated that there was no directional pleiotropy detected, evidenced by *p-*values between 0.117 and 0.845 (Figure 4; Supplementary Table S17). Additionally, a leave-one-out analysis affirmed that the aggregate IVW estimate did not depend on any individual SNP (Supplementary Figure S1).

**Table 5:** Heterogeneity and pleiotropy assessment for significant results (*p* < 0.05).

Exposures	Outcomes	SNPs (*n*)	Directional pleiotropy		Cochran’s *Q* test			Rucker’s *Q*^0^ test			
			Egger intercept	*p*	*Q* statistic	*p*	*I* ^2^	*Q*^0^ statistic	*p*	*Q*–*Q^0^*	*p*
Zinc	Focal epilepsy with HS	5	−0.007	0.117	6.530	0.163	38.720	1.76	0.625	4.770	0.029
Vitamin B6	Focal epilepsy with HS	6	0.003	0.271	3.720	0.591	0	2.09	0.719	1.630	0.202
Vitamin D	CAE	101	0.000	0.641	95.460	0.610	0	95.24	0.588	0.220	0.639
Vitamin D	JAE	101	0.000	0.585	94.823	0.627	0	94.52	0.608	0.303	0.582
Carotene	Focal epilepsy lesion-negative	3	−0.001	0.845	0.080	0.963	0	0.01	0.905	0.070	0.791

## 4 Discussion

In this study, we assessed the causal effects of 15 serum micronutrients on epilepsy using two-sample MR and MVMR. Our findings suggest that vitamin D has a protective effect against CAE and JAE. In contrast, carotenoids were associated with an elevated risk of focal epilepsy with lesion-negativity, but this association was affected by confounders such as zinc and vitamin D. After correction using MVMR, neither vitamin B6 nor zinc showed a significant causal effect on focal epilepsy with HS.

### 4.1 Vitamin B6 and focal epilepsy with HS

Our MR analysis has demonstrated a correlation between vitamin B6 levels and a decreased risk of focal epilepsy with HS, as evidenced by an OR of 0.949, *p* = 0.020. This finding suggests that vitamin B6 may play a protective role in the development of epilepsy, especially in focal types linked to HS. Literature indicates that deficiencies in pyridoxine and its active derivative, pyridoxal-5-phosphate (PLP), are associated with reduced gamma-aminobutyric acid (GABA) levels, leading to seizures and epilepsy manifestations (26–28). A notable aspect of epilepsy pathophysiology is its connection to metabolic and mitochondrial disorders, such as pyridoxine-dependent epilepsy. This condition is characterized by recurrent, intractable neonatal seizures and stems from congenital lysine metabolism errors (29). Remarkably, pyridoxine-dependent epilepsy shows responsiveness to pyridoxine treatment. The essential role of pyridoxine is underscored by its influence on PLP, the bioactive form of vitamin B6 necessary for amino acid synthesis and metabolism. A shortfall in PLP can result in diminished GABA levels in the brain, contributing to the generation of seizures (30–33). To address potential confounding factors of zinc, carotene, and vitamin D, MVMR analysis findings suggest that the initial observed protective effect of vitamin B6 may be influenced by these micronutrients, indicating that the interplay between these micronutrients is complex and warrants further investigation.

While prior studies have shown that elevated pyridoxine levels can aid epilepsy management, aligning with the outcomes presented herein, the significance of PLP extends beyond this context. As a cofactor, PLP is integral to numerous enzymatic processes, facilitating approximately 4% of all known biochemical reactions in the human body (34). Given PLP’s extensive involvement in human physiology, further research is imperative to elucidate the precise impact of pyridoxine on epilepsy.

### 4.2 Vitamin D and CAE/JAE

Our investigations provide compelling evidence linking vitamin D levels to a decreased risk of absence epilepsy, both in childhood and juvenile forms. The findings indicate an OR of 0.976 (95% CI, 0.959–0.993, *p* = 0.006) and 0.986 (95% CI, 0.973–0.999, *p* = 0.032) for both conditions, suggesting a protective role of vitamin D against these epileptic disorders. The MVMR analysis indicated that the causal relationships of vitamin D on CAE/JAE remained. These consistent findings across multiple analyses further validate the protective role of vitamin D against CAE/JAE and emphasize the importance of accounting for potential confounders. The robustness of the results after MVMR adjustment supports the hypothesis that vitamin D plays a crucial role in reducing the risk of CAE and JAE, independent of the influences of zinc, carotene, and vitamin B6.

The protective effect of vitamin D on the risk of childhood and juvenile absence epilepsy, as suggested by the analyses, aligns with vitamin D’s known roles in neural health, including its influence on neuronal growth and differentiation, calcium regulation, and immunomodulation. These functions are critical for maintaining normal neural activity and preventing the hyperexcitability that characterizes epilepsy (35–38).

The consistency of the odds ratios across both childhood and juvenile absence epilepsy enhances the robustness of the association and suggests a potentially universal mechanism of action of vitamin D in modulating epilepsy risk (39, 40). Nevertheless, further research is needed to explore the underlying biological pathways through which vitamin D exerts its protective effect and to determine whether vitamin D supplementation could be a feasible and effective strategy for epilepsy prevention or management.

### 4.3 Zinc and focal epilepsy with HS

Zinc plays a critical role in balancing neuronal excitation and inhibition, impacting various targets. It may significantly influence the pathophysiology of seizures and epilepsy. During neuronal excitation, zinc can be co-released with glutamate from glutamatergic neurons predominantly located in the hippocampus and amygdala (41). The extracellular release of zinc in conditions like epilepsy has been implicated in neurotoxicity (42). Our findings indicate an increased risk of focal epilepsy with HS, associated with zinc, paralleling mechanistic research where zinc ions enhance neuronal excitability and provoke seizures through the activity of the Na^+^–K^+^ ATPase, altering GABA levels or carbonic anhydrase activity (43). Conversely, studies found that oral zinc supplementation served as an adjunct therapy in children with intractable epilepsy, suggesting a dose-dependent dichotomy where zinc can exhibit both neurotoxic and neuroprotective properties (5, 44). The observed OR of 1.010 (95% CI, 1.000–1.020, *p* = 0.045) for zinc’s effect on focal epilepsy with HS from two-sample MR, and MVMR results were 1.010 (95% CI, 1.000–1.020, *p* = 0.057) with carotene, 1.008 (95% CI, 0.998–1.019, *p* = 0.114) with vitamin B6, and 0.998 (95% CI, 0.976–1.020, *p* = 0.837). After correction for MVMR, there was no significant causal relationship. This may be related to the dual role of zinc in the nervous system reported in the literature, and its effect on epilepsy is susceptible to other micronutrient factors.

These findings necessitate a nuanced interpretation, reflecting a fine balance in zinc’s impact on epilepsy risk. This equilibrium underscores the complexity of micronutrient interactions within the neural landscape, emphasizing the importance of comprehensively understanding zinc’s neurobiological effects.

### 4.4 Carotene and focal epilepsy lesion-negative

Our study results indicate a notable correlation between carotene levels and an increased risk of focal epilepsy lesion-negative cases, with an odds ratio (OR) of 1.129 (95% CI: 1.008–1.264, *p* = 0.037). The enzymatic conversion of carotene to retinoic acid, a key molecule in oxidative stress regulation and neuronal excitability modulation, is well-documented (45–47). Despite carotene’s antioxidant reputation, its role in epilepsy, especially discerned in animal studies, warrants further investigation (6, 48). Retinoic acid’s influence on synaptic transmission and its interaction with GABA receptors in hippocampal neurons may be pivotal in seizure mechanisms (49).

The MVMR analysis considered the influence of zinc, and vitamin D on the relationship between carotene and focal epilepsy lesion-negative. After correcting for MVMR, the causal relationship between carotene and focal epilepsy lesion-negative was no longer significant when adjusted for zinc but remained significant with adjustments for vitamin B6 and vitamin D. Interestingly, vitamin D adjustment indicated a protective effect, suggesting carotene’s role might shift depending on the presence of other micronutrients. The MVMR analysis underscores the complexity of micronutrient interactions within the neural landscape and the importance of considering these interactions to avoid confounding effects in epilepsy research. This discovery mandates further research into the intricate relationships between antioxidants, neural activity, and epilepsy, highlighting the necessity for comprehensive biochemical and neurophysiological studies to clarify the link between carotene and epilepsy.

This investigation faced limitations, notably the reliance on GWAS data primarily derived from individuals of European descent. This focus restricts the generalizability of our conclusions across different ethnicities, where varying causal relationships may exist. Furthermore, our analysis was constrained by the inability to differentiate findings by gender and age, as the meta-GWAS did not adjust for these factors, limiting our exploration of potential variances and non-linear associations related to these demographics.

In summary, our study elucidates the complex dynamics between micronutrient levels and epilepsy risk. It delineates a protective influence of vitamin B6 and D against epilepsy while highlighting the nuanced, potentially dose- and context-dependent roles of zinc and carotene in epilepsy etiology. The potential interactions between these micronutrients and their co-impact on epilepsy risk are particularly noteworthy. It accentuates the imperative for detailed and diverse nutritional and neurobiological studies to fully comprehend and harness the potential of micronutrients in mitigating epilepsy risk, paving the way for targeted therapeutic strategies.

## Data availability statement

The original contributions presented in the study are included in the article/Supplementary material, further inquiries can be directed to the corresponding author.

## Author contributions

HC: Data curation, Formal analysis, Investigation, Methodology, Software, Visualization, Writing – original draft. ZZ: Data curation, Investigation, Methodology, Software, Visualization, Writing – original draft. XC: Data curation, Writing – original draft. FG: Conceptualization, Funding acquisition, Project administration, Supervision, Writing – review & editing.

## Footnotes

^1^https://gwas.mrcieu.ac.uk/
