# Supplementary material for: Causal links between serum micronutrients and epilepsy: a Mendelian randomization analysis
Source: Front Neurol. 2024 Jul 15;15:1419289. doi: 10.3389/fneur.2024.1419289 (PMC11284170; doi:10.3389/fneur.2024.1419289)
Supplement: Supplementary file 1 [file Data_Sheet_1.PDF]

Causal Links Between Serum Micronutrients and Epilepsy: A Mendelian Randomization Analysis

Supplementary Figures and Figure Legends

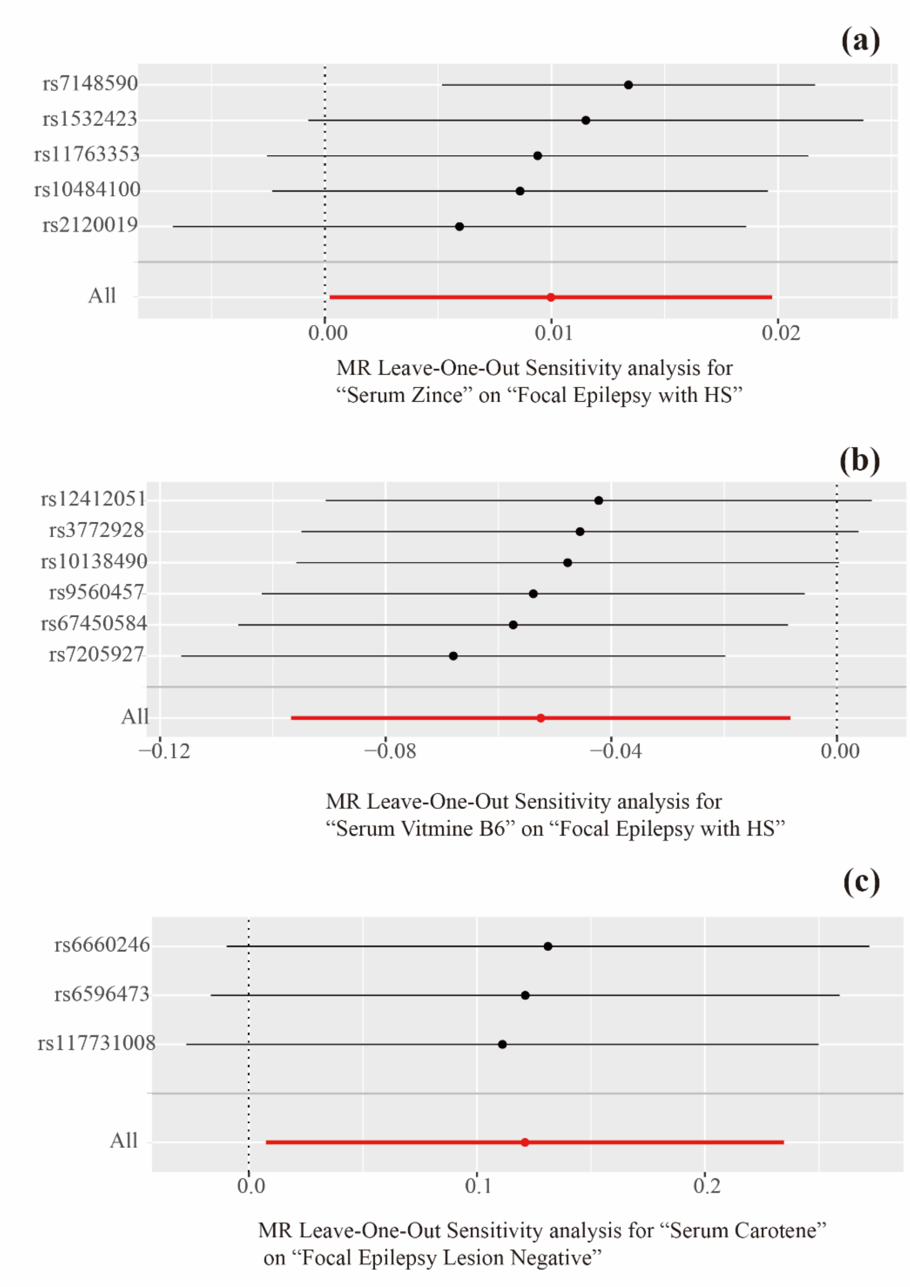

Figure S1: Leave-one-out analyses for robust validation. Each graph displays the effect of sequentially excluding each SNP on the estimated causal relationship between a particular micronutrient and epilepsy phenotype. Black dots depict the causal estimates for each exclusion, while red dots represent the aggregated causal estimates obtained through the IVW approach. Horizontal lines across the dots indicate the 95% confidence intervals for these estimates. (a) Serum zinc on focal epilepsy with HS; (b) Serum carotene on focal epilepsy lesion-negative; (c) Serum vitamin B6 on focal epilepsy with HS. HS, hippocampal sclerosis.
